# Direct conversion of CO_2_ to CH_4_ on Pd/graphdiyne single-crystalline

**DOI:** 10.1093/nsr/nwae189

**Published:** 2024-05-29

**Authors:** Chao Zhang, Xuchen Zheng, Yang Gao, Chengyu Xing, Siao Chen, Yurui Xue, Yuliang Li

**Affiliations:** CAS Key Laboratory of Organic Solids, Institute of Chemistry, Chinese Academy of Sciences, Beijing 100190, China; Shandong Provincial Key Laboratory for Science of Material Creation and Energy Conversion, Science Center for Material Creation and Energy Conversion, School of Chemistry and Chemical Engineering, Shandong University, Jinan 250100, China; CAS Key Laboratory of Organic Solids, Institute of Chemistry, Chinese Academy of Sciences, Beijing 100190, China; Shandong Provincial Key Laboratory for Science of Material Creation and Energy Conversion, Science Center for Material Creation and Energy Conversion, School of Chemistry and Chemical Engineering, Shandong University, Jinan 250100, China; CAS Key Laboratory of Organic Solids, Institute of Chemistry, Chinese Academy of Sciences, Beijing 100190, China; Shandong Provincial Key Laboratory for Science of Material Creation and Energy Conversion, Science Center for Material Creation and Energy Conversion, School of Chemistry and Chemical Engineering, Shandong University, Jinan 250100, China; CAS Key Laboratory of Organic Solids, Institute of Chemistry, Chinese Academy of Sciences, Beijing 100190, China; Shandong Provincial Key Laboratory for Science of Material Creation and Energy Conversion, Science Center for Material Creation and Energy Conversion, School of Chemistry and Chemical Engineering, Shandong University, Jinan 250100, China; CAS Key Laboratory of Organic Solids, Institute of Chemistry, Chinese Academy of Sciences, Beijing 100190, China; Shandong Provincial Key Laboratory for Science of Material Creation and Energy Conversion, Science Center for Material Creation and Energy Conversion, School of Chemistry and Chemical Engineering, Shandong University, Jinan 250100, China; CAS Key Laboratory of Organic Solids, Institute of Chemistry, Chinese Academy of Sciences, Beijing 100190, China; School of Chemical Sciences, University of Chinese Academy of Sciences, Beijing 100049, China; CAS Key Laboratory of Organic Solids, Institute of Chemistry, Chinese Academy of Sciences, Beijing 100190, China; Shandong Provincial Key Laboratory for Science of Material Creation and Energy Conversion, Science Center for Material Creation and Energy Conversion, School of Chemistry and Chemical Engineering, Shandong University, Jinan 250100, China

**Keywords:** graphdiyne, controllable growth, CO_2_ conversion, single-crystalline, heterocatalysis

## Abstract

A major impediment to the development of the efficient use of artificial photosynthesis is the lack of highly selective and efficient photocatalysts toward the conversion of CO_2_ by sunlight energy at room temperature and ambient pressure. After many years of hard work, we finally completed the synthesis of graphdiyne-based palladium quantum dot catalysts containing high-density metal atom steps for selective artificial photosynthesis. The well-designed interface structure of the catalyst is composed of electron-donor and acceptor groups, resulting in the obvious incomplete charge-transfer phenomenon between graphdiyne and plasmonic metal nanostructures on the interface. These intrinsic characteristics are the origin of the high performance of the catalyst. Studies on its mechanism reveal that the synergism between ‘hot electron’ from local surface plasmon resonance and rapid photogenerated carrier separation at the ohmic contact interface accelerates the multi-electron reaction kinetics. The catalyst can selectively synthesize CH_4_ directly from CO_2_ and H_2_O with selectivity of near 100% at room temperature and pressure, and exhibits transformative performance, with an average CH_4_ yield of 26.2 μmol g^−1^ h^−1^ and remarkable long-term stability.

## Teaser

A Pd/graphdiyne quantum dot catalyst containing high-density metal atom steps was controllably synthesized for efficient artificial photosynthesis at ambient conditions with 100% CO_2_-to-CH_4_ conversion selectivity.

## INTRODUCTION

Artificial photosynthesis (AP), which converts carbon dioxide (CO_2_) into high-value chemicals/fuels by harvesting sunlight energy at room temperature and ambient pressure, has been a scientific dream of humanity for many years. However, with the passage of time and the discovery of highly selective and efficient photocatalysts, more and more researchers are seeing the light at the end of the tunnel of this scientific dream. The AP process highly depends on the reaction selectivity and efficiency of photocatalysts [1–17]. Metastable nanomaterials with excitated local surface plasmon resonance (LSPR) properties can enable enhanced light absorption and activate chemical bonds near the metal surface, providing an effective method for selective and efficient solar-to-chemical conversion [18–22]. Recently, various LSPR-type quantum dot (QD) catalysts have been developed to improve the efficiency of various photocatalytic processes. However, the unstable active site, inert surface and rapid carrier recombination of reported catalysts seriously affect their large-scale applications [23–27]. A new idea is to anchor metals on high conjugated support materials to efficiently promote charge separation, avoid the aggregation of the active sites, and generate transformative catalytic properties.

Graphdiyne (GDY) with sp/sp^2^-cohybridized carbon atoms has shown numerous natural advantages when it comes to resolving the drawbacks of traditional catalysts, such as the large π conjugated networks, natural pores, intrinsic band gap, infinite active sites, high carrier mobility, tunable electronic properties and excellent stability [28–40]. In particular, the special cavity structure of GDY allows it to control the anchoring of single or multiple atoms, which eventually gradually grow into QDs and clusters. This growth process in the GDY cavity can effectively avoid the aggregation of active sites under continuous irradiation, thus guaranteeing the activity and stability of the catalysts. Moreover, electrons in acetylenic linkages of GDY are prone to delocalization, resulting in a distinctive interface structure with optimized dielectric environment [36,39]. These properties are very conducive to the synthesis of photocatalysts with excellent reaction selectivity, activity and stability. It is very valuable to use LSPR and GDY for AP to break through the great challenges encountered in traditional light synthesis and produce transformative effects.

In this study, we report a facile strategy for the controllable synthesis of well-defined Pd/graphdiyne single-crystalline with high-density metal atom steps on GDY for CO_2_RR. Experimental results show that the unique structures of the catalyst can result in the strong incomplete charge-transfer phenomenon between GDY and Pd QDs and accelerated charge-transfer dynamics leading to transformative photocatalytic performances. Studies on the mechanism show that bridge-bonded CO on the Pd QD edge/corners are the active sites for selective hydrogenation of CO_2_, and the ohmic contact between GDY and Pd QDs originated from sp-C-Pd bonds efficiently accelerates the charge-transfer dynamics. Benefiting from these unique features, Pd QDs/GDY reaches high AP performances with near 100% selectivity for CH_4_, and average yields of 26.2 μmol g^−1^ h^−1^ at room temperature and ambient conditions.

## RESULTS AND DISCUSSION

**Figure 1.**
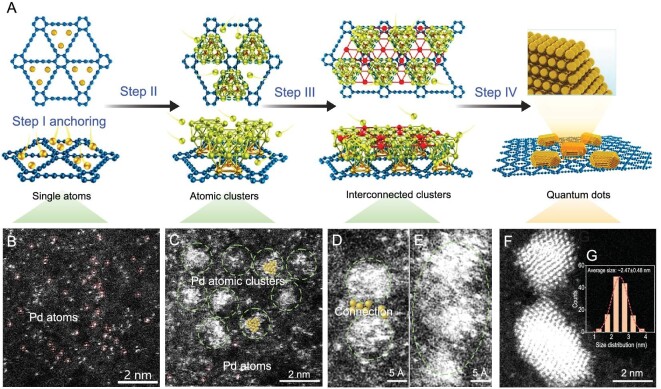
Synthesis of Pd QDs/GDY. (A) Schematic route to the synthesis of Pd QDs/GDY. (B–F) HAADF-STEM images of the samples obtained at different stages from (B) single Pd atoms to (C) Pd atoms + clusters and (D–F) Pd QDs. (G) Size distribution of Pd QDs/GDY (>130 Pd QDs were counted).

**Figure 2.**
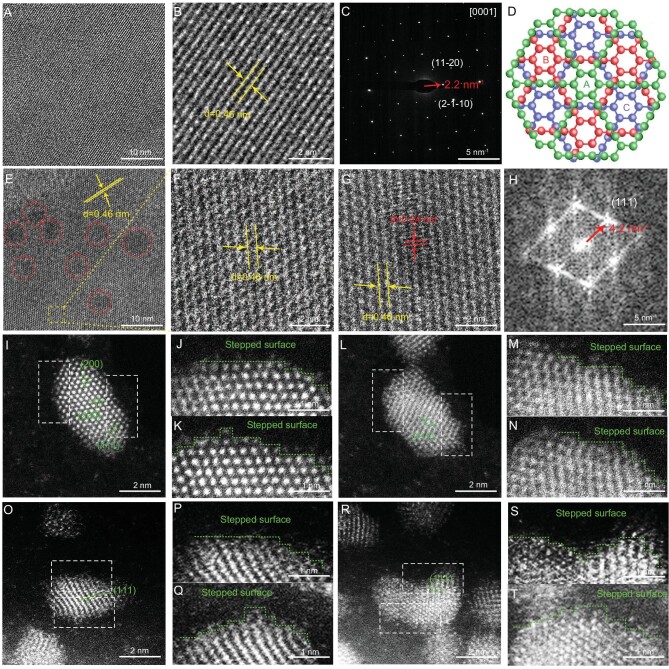
Morphological characterizations. (A and B) HRTEM images of GDY. (C) Selected area electron diffraction (SAED) patterns of GDY. (D) ABC-stacking configuration of graphdiyne (top view). (E and F) HRTEM images of Pd QDs. (G) SAED patterns of Pd QDs. (H) Fast Fourier transform (FFT) patterns of Pd QDs. (I–T) HAADF-STEM images of Pd QDs/GDY. (J and K) Enlarged HAADF-STEM images of metal atom steps of the marked areas at left and right sides in (I). (M and N) Enlarged HAADF-STEM images of metal atom steps of the marked areas at left and right sides in (L). (P and Q) Enlarged HAADF-STEM images of metal atom steps of the marked areas at upper and lower sides in (O). (S and T) Enlarged HAADF-STEM images of metal atom steps of the marked areas at upper and lower sides in (R).

The catalysts were firstly synthesized through a micro-interface-induced assembly coupling process (Fig. 1A), including the first formation of alkyne-Pd complexes through the reaction between palladium species and hexaethynylbenzene (HEB), and the following alkyne-alkyne coupling reaction leading to the successful synthesis of graphdiyne and the simultaneous anchoring of Pd atoms on GDY (Step I) as evidenced by the sub-Ångström-resolution aberration-corrected scanning transmission electron microscopy (HAADF-STEM, Fig. 1B). As the reaction time increased, Pd atom clusters (Fig. 1C, Step II) were gradually formed, and connected together to form larger sizes (Fig. 1D and E; Step III), finally resulting in Pd QDs (Fig. 1F, Step IV, sizes: 2.47 ± 0.48 nm; Fig. 1G) [41,42]. High-resolution transmission electron microscopy (HRTEM) images show that the synthesized GDY is all-crystalline with the lattice fringe of 0.46 nm (Fig. 2A and B) indexed by the (11–20) reflections of GDY (Fig. 2C) and the ABC-stacking mode (Fig. 2D) [43,44]. The lattice spacings of 0.46 nm (Fig. 2E and F) and 0.24 nm (Fig. 2G and H) corresponding to GDY and Pd QDs were all observed, indicating the successful synthesis of Pd/GDY crystalline, and demonstrating the excellent structural stability of the sample. The Pd QDs were evenly distributed on the surface of single-crystalline GDY. Energy-dispersive spectroscopy (EDS, Fig. S4) elemental mapping results confirm the uniform distribution of Pd and C elements in the samples. HAADF-STEM characterizations show that the (111), (200) and (220) planes of Pd were observed from Fig. 2I. Large numbers of atomic steps were formed at the edge of the (111) plane of Pd QDs (Fig. 2J–T), exposing high-density atomic sites on the surface of Pd QDs, which are supposed to be more active than those in bulk catalysts and bring higher intrinsic activities.

**Figure 3.**
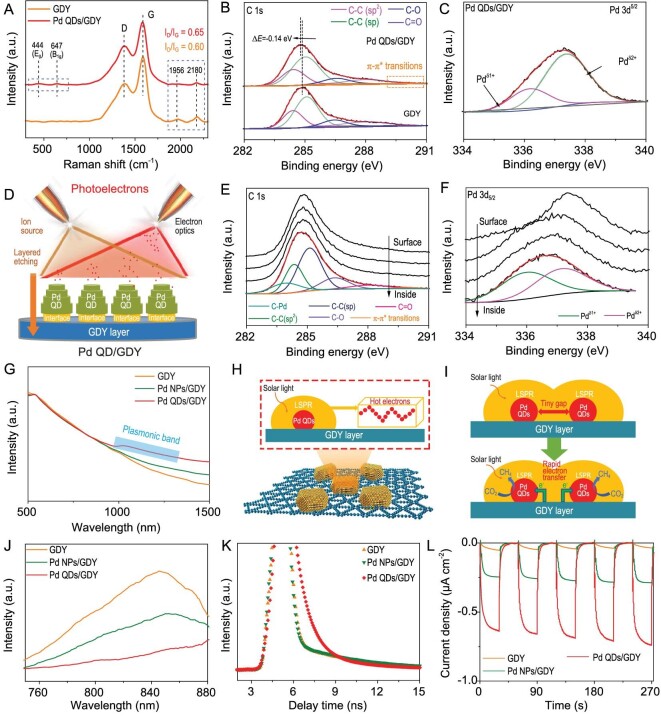
Electronic and photonic properties of the samples. (A) Raman spectra of Pd QDs/GDY and references. (B and C) C 1s and Pd 3d_5/2_ XPS spectra of Pd QDs/GDY and GDY. (D) Schematic representation of the depth profiling experiments. (E and F) C 1s and Pd 3d_5/2_ XPS spectra of Pd QDs/GDY from the depth profiling experiments. (G) UV-DRS results of Pd QDs/GDY and references. (H and I) Schematic diagram of the plasmonic heterostructure with numerous active ‘hot spots’ for photocatalytic CO_2_ reduction. (J) Steady-state photoluminescence (PL) spectrum of Pd QDs/GDY and references. (K) Transient PL decay curves of Pd QDs/GDY and references. (L) Amperometry *i–t* curves at 0 V vs. Ag/AgCl in 0.1 M Na_2_SO_4_ solution under illumination.

The structures of the catalysts were next characterized by Raman, X-ray photoelectron spectroscopy (XPS) and UV-visible diffuse reflectance spectrum (UV-DRS). Raman spectra (Fig. 3A) of the Pd QDs/GDY show characteristic peaks of D band (1378 cm^−1^), G band (1582 cm^−1^) and conjugated acetylene links (1956 and 2180 cm^−1^) and the survey XPS (Fig. S6) confirm the successful synthesis of Pd QDs/GDY [37,38]. High-resolution C 1s XPS spectra (Fig. 3B and Fig. S7) of GDY and its heterostructure can be deconvoluted into four characteristic sub-peaks of C–C(sp^2^), C–C(sp), C–O and C=O, respectively [36,39]. An additional peak corresponding to π–π* transition newly appeared in Pd QDs/GDY, reflecting the strong interaction between Pd and GDY (Fig. S8). Pd 3d_5/2_ XPS spectra of Pd QDs/GDY (Fig. 3C) can be divided into two sub-peaks at 336.2 and 337.4 eV, which are noted as Pd^δ1+^ and Pd^δ2+^, respectively. The binding energy of Pd^δ2+^ implies the intermediated valence between Pd^2+^ and Pd^4+^ (337.8 eV). In Pd 3d_5/2_ XPS spectra of Pd QDs/GDY, peaks at 336.2 and 337.4 eV were recorded and assigned to the Pd^0^ and positively charged Pd (Pd^δ+^), respectively (Fig. 3C) [43–51]. Depth-profiling XPS coupled with ion sputtering (Fig. 3D–F and Fig. S9) shows an obvious sub-peak at 283.9 (sp-C-Pd), while no additional peaks can be identified for the Pd nanoparticles (NPs)/GDY samples [52]. These results originated from the strong intercalations between Pd and GDY to form a sp-C–Pd bond, which can stabilize Pd QDs and eliminate the charge-transfer barriers. Figure 3G exhibits an obvious plasmon peak that might be due to the strong plasmonic coupling between Pd QDs and GDY [25,26]. The unique tiny intervals between adjacent Pd QDs (Fig. S10) can significantly accelerate the generation of ‘hot electron’ (Fig. 3H and I) [2,45], and promote charge separation evidenced by the steady-state photoluminescence (PL) emission spectrum (Fig. 3J) and transient PL decay curves (Fig. 3K and Table S2). The electrochemical impedance spectroscopy (EIS, Figs S11 and S12 and Table S3) results show that Pd QDs/GDY has lower charge-transfer resistance (*R*_ct_, 574 Ω) than GDY (2421 Ω) and Pd NPs/GDY (1255 Ω), confirming its accelerated charge transport ability. Moreover, Pd QDs/GDY exhibits a higher transient photocurrent (0.74 μA cm^−2^) than GDY (0.03 μA cm^−2^) and Pd NPs/GDY (0.29 μA cm^−2^), further revealing the excellent photogenerated carrier separation capacity (Fig. 3L).

**Figure 4.**
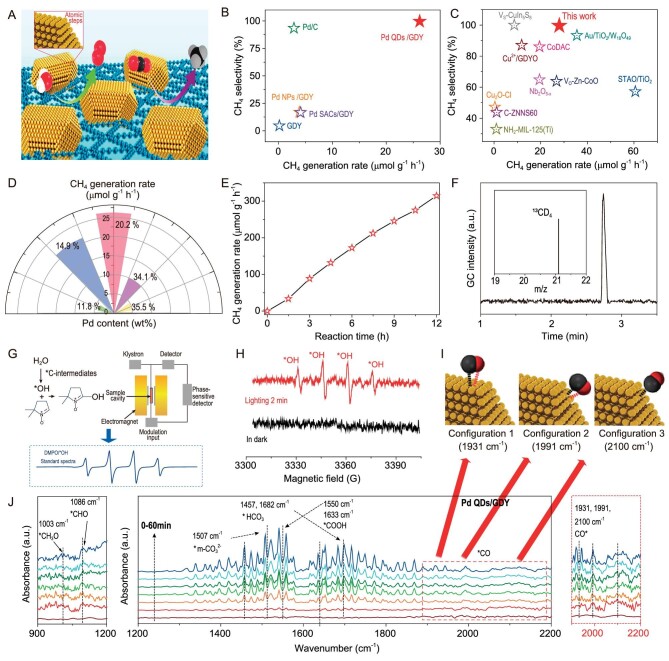
Photocatalytic performance measurements. (A) Schematic illustration of the photocatalytic process. (B) The CH_4_ selectivity and CH_4_ generation rate for the catalysts. (C) Comparison of the photocatalytic performances of Pd QDs/GDY with reported catalysts. (D) Relation between CH_4_ generation rates and the mass loading of Pd in Pd QDs/GDY. (E) The variation of the CH_4_ generation rate along with the reaction time. (F) Mass spectrometry (MS) results of ^13^CD_4_ (m/z = 21.1) converted from ^13^CO_2_ and D_2_O. (G) Schematic diagram of the mechanism of EPR applied to hydroxyl radical detection. (H) EPR results for DMPO before and after artificial photosynthesis. (I) Three configurations of CO adsorbed on Pd QDs/GDY. (J) *In-situ* DRIFTS results of Pd QDs/GDY during the artificial photosynthesis process.

The photocatalytic activities of the synthesized catalysts for CO_2_ reduction were evaluated using an internal gas circulation system (Beijing Perfectlight, Labsolar 6A) under solar irradiation at the solid–gas interface, without the addition of any organic sacrificial reagents (Fig. 4A). The transformed gaseous products were analyzed using a gas chromatograph (Agilent 7890 B) with an online injection system utilizing an external standard method (standard gas data are shown in Fig. S13), and liquid products were determined by nuclear magnetic resonance (NMR). High-purity carbon dioxide was used as the gas source (Fig. S14). A series of control experiments were conducted under variable conditions (Figs S15 and S16: normal condition, without CO_2_, without irradiation, without photocatalysts), demonstrating that the detected products all come from the photo-conversion of CO_2_ and no liquid products could be determined (Fig. S17). Remarkably, Pd QDs/GDY shows 100% reaction selectivity towards the CO_2_-to-CH_4_ conversion (Fig. 4B) and a high CH_4_ yielding rate (Y_CH4_) of 26.2 μmol g^−1^ h^−1^ (Fig. 4C). These results are superior to commercial 5% Pd/C and reported catalysts (Fig. 4B–D and Table S4). In contrast, GDY and Pd NPs/GDY had very low CH_4_ selectivity. Pd QDs/GDY exhibits wonderful stability, as represented by the tiny decrease in yield after continuous work for >12 hours (Fig. 4E). This again indicates that the excellent catalytic performance originated from the specific interactions between Pd QDs and GDY. Isotope-labeling experiments (^13^CO_2_ and D_2_O) confirm that the carbon and hydrogen elements in CH_4_ come from CO_2_ and H_2_O, respectively (Fig. 4F). The water-splitting processes in artificial photosynthesis have also been demonstrated by electron paramagnetic resonance (EPR) with 5,5-dimethyl-1-pyrroline *N*-oxide (DMPO) as the free radical trapping agent (Fig. 4G). An apparent EPR signal of *OH after 2-minute irradiation suggests the high charge-separation efficiency of Pd QDs/GDY (Fig. 4H and Fig. S18) [53].

**Figure 5.**
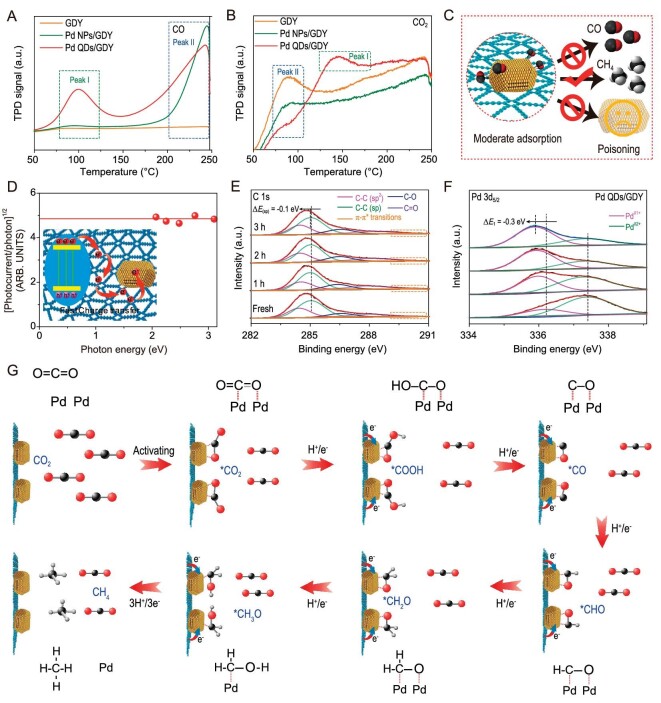
Structural evolution during photocatalysis. (A) CO temperature-programmed desorption (CO-TPD) of Pd QDs/GDY and references. (B) Temperature-programmed desorption (CO_2_-TPD) of Pd QDs/GDY and references. (C) Schematic diagram of adsorption behaviors on Pd QDs/GDY. (D) Linear fitting of the normalized photocurrent to the photon energy of Pd QDs/GDY (inset: charge transfer illustration of the catalyst). (E, F) High-resolution (E) C 1s and (F) Pd 3d_5/2_ XPS spectra of Pd QDs/GDY before and after photo-conversion. (G) Possible reaction paths of photo-conversion on Pd QDs/GDY.

*In-situ* diffuse reflectance infrared Fourier transform spectroscopy (DRIFTS, Fig. 4J and Fig. S19) shows the characteristic peaks of chelating-bridged carbonate (m-CO_3_^2−^, 1512 cm^−1^) [54,55], and symmetric/asymmetric stretching of adsorbed HCO_3_^−^ (1650 cm^−1^) [22,56], COOH* (1568 cm^−1^) and *CO_2_ (1697 cm^−1^), respectively during the reaction process [22,57,58]. The peaks at 1931, 1991 and 2100 cm^−1^ can be attributed to bridge-bonded CO on the Pd(111) facets (Configuration 1) and the edge/corners of Pd QDs (Configuration 2), and linear CO on Pd(111) facets (Configuration 3), respectively [59], as shown in Fig. 4I. The formation of methane during the reaction was also evidenced by the peaks at 1003 and 1086 cm^−1^ corresponding to the *CH_3_O and *CHO species [22,60]. Temperature programmed desorption of CO (CO-TPD) measurement (Fig. 5A) shows two obvious desorption signals on Pd QDs/GDY, which originate from bridge-bonded CO on the Pd QDs atomic steps (Configuration 2) and linear CO on Pd (111) facets (Configuration 3), respectively. Considering that Configuration 1 is more stable than Configuration 3, the fact that no signal of Configuration 1 can be detected in the CO-TPD measurement might be due to the stronger adsorption of CO in Configuration 1 than in Configuration 3. This phenomenon also reveals the weaker adsorption strength of CO on the edge/corners than that on Pd (111) facets, which benefits subsequent reactions. CO_2_-TPD measurement (Fig. 5B) shows that Pd QDs/GDY has stronger CO_2_ adsorption capacity than GDY, benefitting from the complete hydrogenation. Considering the selectivity difference in the photocatalytic tests, it can be summarized that the high-density atomic sites on the surface of Pd QDs/GDY have the moderate adsorption ability that is important for hydrogenation of CO (Fig. 5C).

Ultraviolet electron spectroscopy (UPS, Figs S20 and S21 and Table S5) shows decreased Fermi energy (*E*_Fermi_) in the heterostructure, suggesting charge transfer from GDY to Pd QDs at the heterogeneous interface. The linear fitting of the normalized photocurrent to the photon energy (Fig. 5D and Fig. S22) shows the ohmic contact between GDY and Pd QDs. The Schottky barrier between GDY and Pd NPs is ∼1.61 eV [61,62]. This obvious difference can be attributed to the discrepant chemical composition at the heterogeneous interface. As shown in Fig. 5E, there are no changes for sp2-C peaks during the catalysis process, while the sp-C peaks shift to lower binding energies as the reaction proceeds. The shift of C 1s XPS peak indicates the transfer of photogenerated electrons to sp-C of GDY under continuous irradiation. High-resolution Pd 3d_5/2_ of Pd QDs/GDY spectra (Fig. 5F) show the obvious negative shift in binding energies for Pd (δ1+), which indicates that Pd (δ1+) as electron acceptors obtains numerous electrons from GDY during artificial photosynthesis. The electron migration (Fig. 5E and F) in Pd QDs/GDY demonstrated that the photogenerated electrons rapidly transferred from GDY to Pd QDs, and the hot electrons from ‘hot spots’ are simultaneously injected into CO_2_ for reduction. According to the *in-situ* experimental results and photo-conversion behaviors, possible reaction paths in Pd QDs/GDY for artificial photosynthesis are demonstrated (Fig. 5G). Firstly, electron-rich Pd QDs capture bridge-bonded CO_2_ via high-density atomic steps and further activate it to *CO_2_. With the presence of H^+^, *CO_2_ can then be transformed to *COOH and further forms *CO through dehydration. Bridge-bonded CO is subsequently hydrogenated to form *CHO on Pd QDs instead of CO releasing or catalyst poisoning through the accelerated charge transfer. The obtained *CHO undergoes continuous hydrogenation and dehydration to form CH_4_.

## CONCLUSIONS

In summary, we have developed a micro-interface-induced assembly coupling strategy to achieve the successful synthesis of a crystalline-graphdiyne-based plasmonic heterostructure for efficient photo-conversion of CO_2_. A series of structural characterizations such as HRTEM, HAADF-STEM and XPS demonstrate the precise structure of the Pd QDs/GDY catalyst. The experimental results confirm that the incomplete charge transfer between donors of GDY and acceptors of Pd QDs, and the enhanced LSPR, greatly improve the reaction path and separation efficiency of photogenerated carriers. *In-situ* DRIFTS, CO-TPD and *in-situ* XPS results confirm that the synergism of high-energy electron generation and separation promotes the efficient conversion of CO_2_ at atomic steps in Pd QDs. As a result, Pd QDs/GDY exhibits superior selectivity (∼100%) and outstanding photocatalytic activity (up to 26.2 μmol g^−1^ h^−1^) under normal conditions. This work provides new insights into the design and synthesis of plasmonic metal nanostructures for selective artificial photosynthesis, which is a step towards the next generation of catalytic systems for photosynthesis.

## Supplementary Material

nwae189_Supplemental_File
