# Supplementary material for: Direct conversion of CO2 to CH4 on Pd/graphdiyne single-crystalline
Source: Natl Sci Rev. 2024 May 29;11(8):nwae189. doi: 10.1093/nsr/nwae189 (PMC11242443; doi:10.1093/nsr/nwae189)
Supplement: nwae189_Supplemental_File [file nwae189_supplemental_file.docx]

Supplementary Information for

**Direct conversion of CO_2_-to-CH_4_ on Pd/graphdiyne single-crystalline** Chao Zhang^1,3^, Xuchen Zheng^1,3^, Yang Gao^1,3^, Chengyu Xing^1,3^, Siao Chen^1,3^, Yurui Xue^1,2*^, Yuliang Li^1,3,4*^

^1^CAS Key Laboratory of Organic Solids, Institute of Chemistry, Chinese Academy of Sciences, Beijing 100190, P. R. China.

^2^Shandong Provincial Key Laboratory for Science of Material Creation and Energy Conversion, Science Center for Material Creation and Energy Conversion, School of Chemistry and Chemical Engineering, Shandong University, Jinan 250100, P. R. China.

^3^University of Chinese Academy of Sciences, Beijing 100049, P. R. China.

^4^Lead Contact for the paper

***Corresponding authors.** E-mails: yrxue@sdu.edu.cn (Y.X.); ylli@iccas.ac.cn (Y.L.).

**1.Experimental Section**

**Materials.**

Sodium palladium tetrachloride (Na_2_PdCl_4_), hydrated cupric chloride (CuCl_2_ 2H_2_O) were purchased from Energy Chemical. Other organic solvents were supplied by Concord Technology. Ar (≥ 99.99%) was obtained from Beijing Zhongke Tailong Electronic Technology Co., Ltd. CO_2_ (≥ 99.999%) was obtained from Beijing Huanyu Jinghui Jingcheng Gas Technology Co., Ltd.

All the reagents and solvents were used without further purification.

**Methods**

**Synthesis of Pd QDs/GDY.**

Pd QDs/GDY was synthesized through a modified Eglinton coupling reaction. 40 mg Na_2_PdCl_4_ was dissolved in 100 ml DI-water (Solution A) and heated to 80 ℃ under Ar atmosphere. Then, 20 mg HEB was dissolved in 50 ml pyridine and dropped in Solution A. After completely drops of Solution A, this reaction system maintained 48 h for sufficient reaction under Argon atmosphere. Pd QDs/GDY was obtained by suction filtration and washed with tetrahydrofuran and water, respectively. Finally, the prepared sample was dried in vacuum for 12 h at 80 ℃.

**Synthesis of crystalline graphdiyne (GDY).**

GDY was prepared under similar procedures with Pd QDs/GDY. 50 mg CuCl_2_ 2H_2_O was dissolved in 100 ml DI-water (Solution A) and heated to 80 ℃ under Ar atmosphere. Then, 20 mg HEB was dissolved in 50 ml pyridine and slowly dropped in Solution A. After completely drops of Solution A, this reaction system maintained 48 h for sufficient reaction under Argon atmosphere. Finally, final products were collected through suction filtration and further treated with 1M HCl, DI-water and tetrahydrofuran. Similarly, the prepared sample was dried in vacuum for 12 h at 80 ℃.

**Synthesis of Pd NPs/GDY.**

7 mg Na_2_PdCl_4_ was dissolved in 10 ml DI-water to form a clear solution. Then this solution was placed in an ice water bath to maintain low temperature. After sufficient cooling, 10 mg prepared GDY was added in it with vigorous stirring. After maintaining for 30 min, Pd NPs/GDY was obtained by suction filtration and washed with water and tetrahydrofuran. Finally, the prepared sample was dried in vacuum for 12 h at 80 ℃.

**Metal content characterization.**

Metal content was determined using inductively coupled plasma analysis (Agilent ICPOES730). Before measurement, samples were digested in a 1:3:4:100 (by weight) mixture of HNO_3_: HCl: HF: H_2_O. The element content in the tested sample is calculated as follows:

$$\omega\left( wt\% \right)=\frac{Conc.\times V}{m\left( cat \right)}\times100\%$$

where Conc. V, and m(cat) are the metal concentration in the mixed solution (mg L^-1^), the volume of solution (L) and the weight of catalyst (g).

**Morphological measurements.**

The morphologies of the samples were recorded through scanning electron microscopy (SEM, Hitachi Model S-4800), transmission electron microscopy (TEM, JEM-2100F) and atomic force microscope (AFM, Bruker FASTSCAN BIO with a sharp Si_3_N_4_ probe).

**High-density atomic defects characterization.**

The high-density atomic steps of the samples were identified by Sub-Ångström-resolution, aberration-corrected scanning transmission electron microscopy (STEM) at atomic resolution high-angle annular dark-field (HAADF). The elements distribution in samples were obtained using STEM–energy-dispersive X-ray spectroscopy (STEM–EDX).

**Composition Characterization.**

Structural information was gained by Renishaw-2000 Raman spectrometer at 473 nm length. X–ray photoelectron spectroscopy and depth-profiling X-ray photoelectron spectroscopy (XPS) coupled with ion sputtering was conducted by Thermo Scientific ESCALab 250Xi instrument under 200-W monochromated Al Kα radiation. Argon ions were used to etch the surface. All binding energies were corrected using carbon element (C 1 s = 284.4 eV).

**Optical properties Characterization.**

UV-Visible absorption measurement was conducted by HITACHI UH4150 UV−Visible spectrophotometer. Fluorescence emission spectra was obtained from FluoroMax under 400 nm excitation. The photoluminescence decay spectra were recorded from Edinburgh FLS980 under laser light source excitation at 405 nm.

The average fluorescence lifetime is gained through formula below:

$$\tau_{average}=\frac{\sum A_{i}\tau_{i}^{2}}{\sum A_{i}\tau_{i}}$$

**Evaluation of gas phase photocatalytic products.**

The photocatalytic experiment was evaluated in an internal gas circulation system (Beijing Perfectlight, Labsolar 6A) through an external standard method. All photocatalysts were pre-heated to 150 ℃ under vacuum for one hour to ensure the accuracy. For each experiment, 5 mg photocatalysts were uniformly dispersion on a quartz glass (3.14 cm^2^) and 10 ml DI-water was added into this system as the photocatalytic reducing reagent. Before each photocatalytic measurements, the system was continuously vacuumized and further injected with high-purity carbon dioxide (≥99.999%). This procedure is repeated three times to ensure the purity of carbon dioxide in the catalytic system. A 300 W Xe-lamp (PLS-SXE300) was used as the light source. Circulating condensation was used to maintain ambient temperature (10 ℃). The final gas products were analyzed through online GC-MS (Agilent 7890B) with an FID detector. Meanwhile, the hydrogen release of samples was recorded on GC 9790Ⅱ (Fuli) with an TCD detector through offline injection.

**Evaluation of liquid phase photocatalytic products.**

Liquid products were further recorded on 1H NMR on a Bruker Avance III 400 HD spectrometer through an internal standard method. The detection solution was prepared in proportion of 500 μl photolysis solution and 100 μl D_2_O mixed with 10 μl dimethyl sulfoxide (DMSO) as an internal. (Standard chemical shift: H_2_O: ~ 4.8 ppm, DMSO: ~2.8 ppm).

**Photoelectrochemical characterizations.**

All photoelectrochemical results were recorded in 0.1 M Na_2_SO_4_ (pH=6.8) through a typical three-electrode electrochemical workstation (CHI. 760D, Shanghai CH. Instruments, China) with the as-prepared photoelectrode, graphite plate, saturated calomel electrode (SCE) as the working electrode, counter electrode and reference electrode, respectively.

**Hydroxyl radical (•OH) determination.**

10 mg samples were dispersed in 2 ml of H_2_O containing 20 μL 5,5-dimethyl-1-pyrroline N-oxide (DMPO) to form the detection solution. The system was then continuously illuminated for 2 min with CO_2_ continuous injection. Then, the hydroxyl radical in solution was detected through electron spin resonance measurement (EPR) with the supernatant.

**Adsorption tests.**

Physical adsorption tests (CO_2_-BET) were measured by ASAP 2020HD88. Chemisorption tests (CO_2_-TPD and CO-TPD) were obtained from AutoChem II2920. Vacuum degassing process at 200 ℃ were implemented before corresponding tests. In-situ FITR measurements were conducted by TENSOR-27 with corresponding attachments.

**Ultraviolet photoelectron spectroscopy (UPS) tests.**

Ultraviolet photoelectron spectroscopy was recorded by Thermo Scientific ESCALab 250Xi instrument. The UPS results are gained through formula below:

Φ = hν − E_Fermi_ + E_cutoff_

where hν, E_Fermi_ and E_cutoff_ are utilized photoenergy, Fermi level edge and inelastic secondary electron cutoff, respectively.

**2. Supplementary Figures**


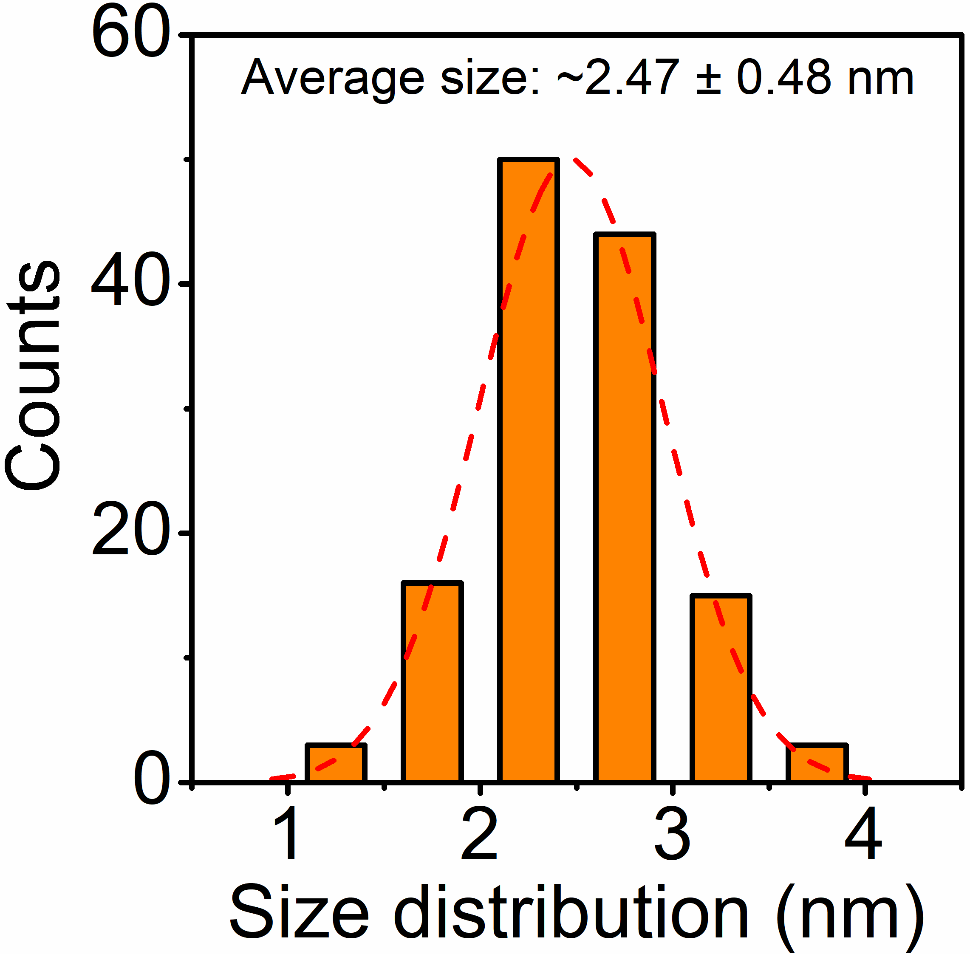


**Supplementary Figure S1.** Size distribution of Pd QDs/GDY (>130 Pd QDs were considered).


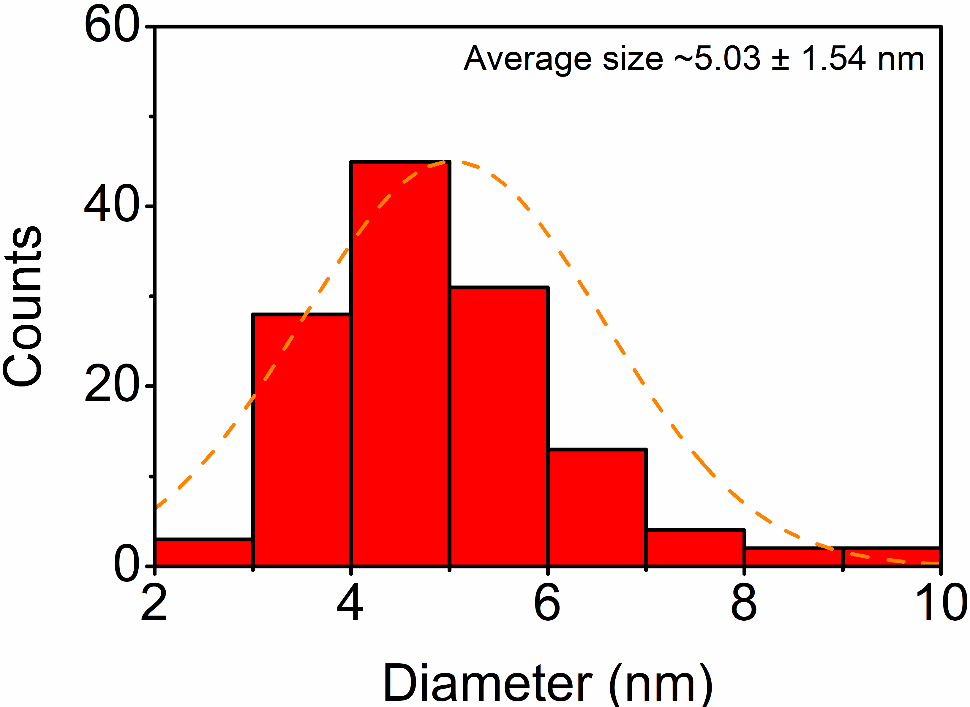


**Supplementary Figure S2**. Size distribution of Pd NPs/GDY (>130 Pd NPs were counted).


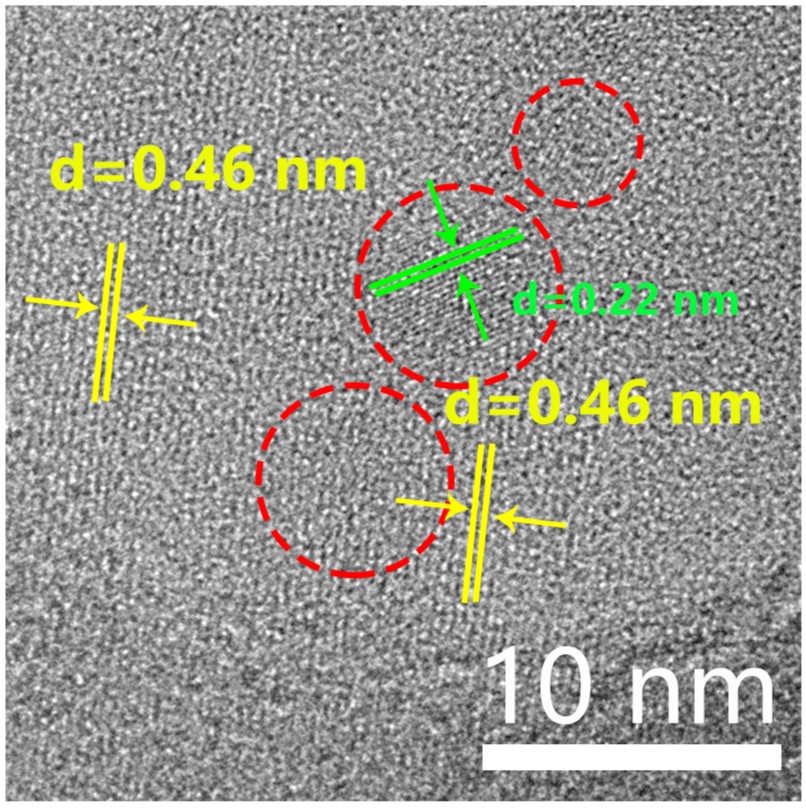


**Supplementary Figure S3**. HRTEM image of Pd NPs/GDY.


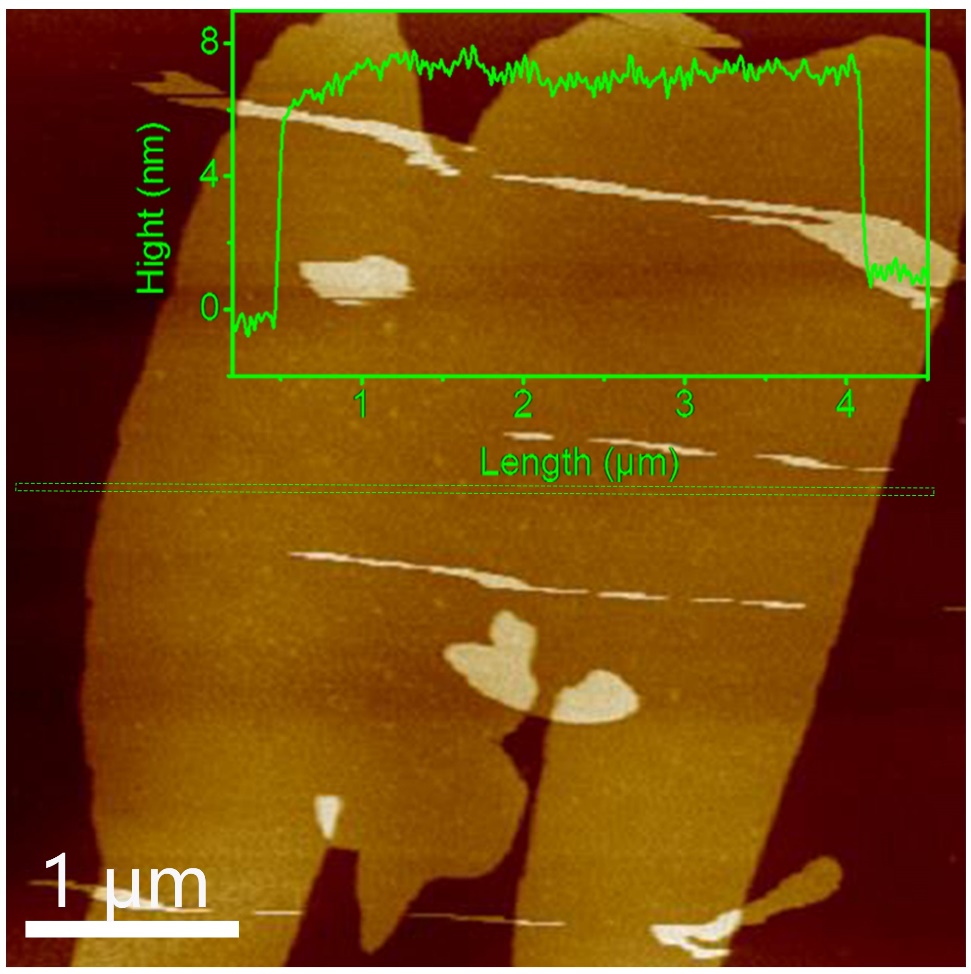


**Supplementary Figure S4.** AFM image of Pd QDs/GDY.


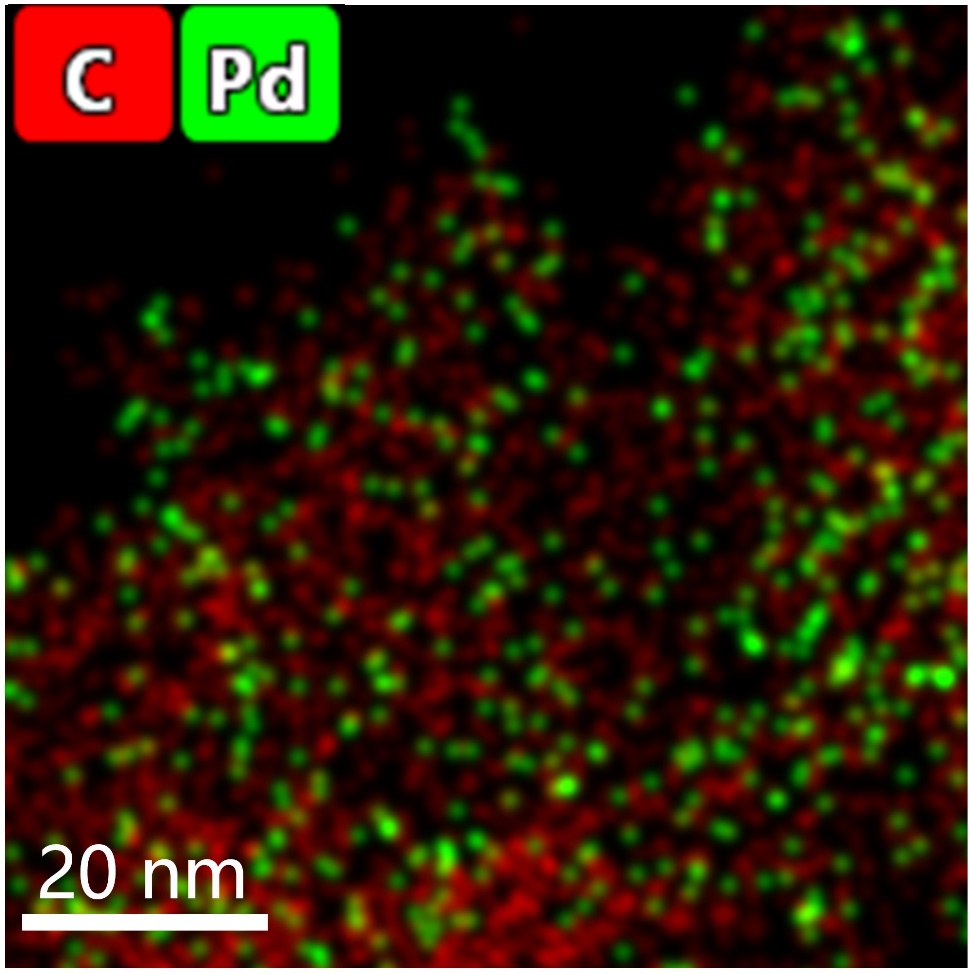


**Supplementary Figure S5.** Elemental mapping image of Pd QDs/GDY.


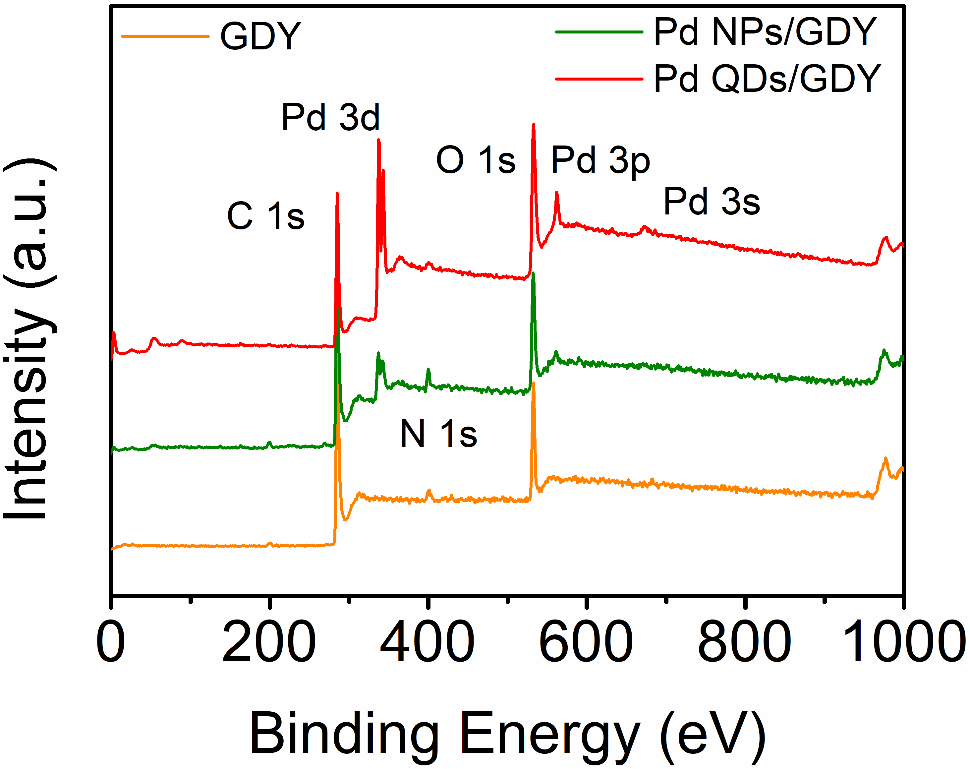


**Supplementary Figure S6.** XPS survey spectra of Pd QDs/GDY, Pd NPs/GDY and GDY.


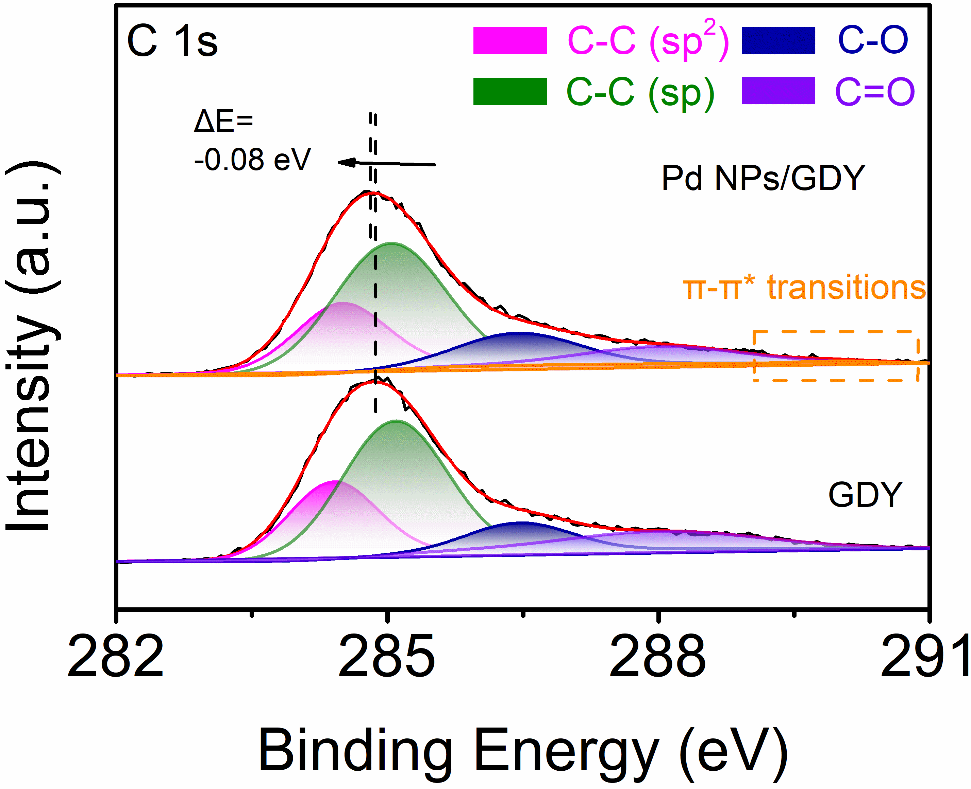


**Supplementary Figure S7.** C1s XPS spectra of Pd NPs/GDY and GDY.


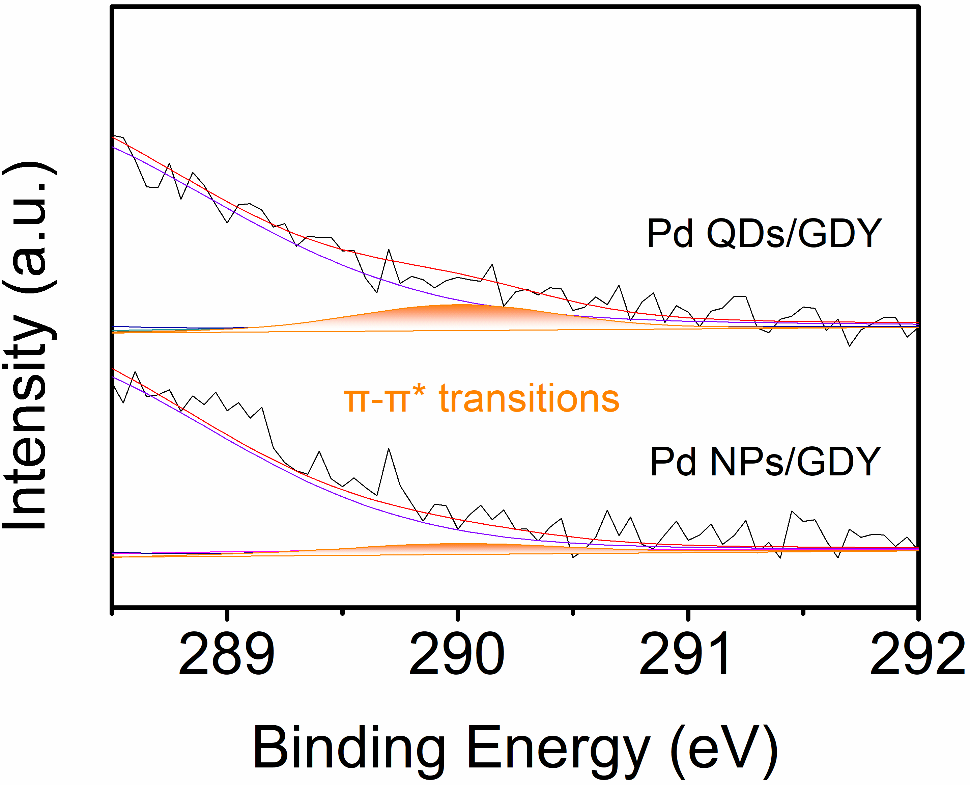


**Supplementary Figure S8.** XPS detailed π-π* satellite peak of Pd QDs and Pd NPs/GDY of C 1s orbital.


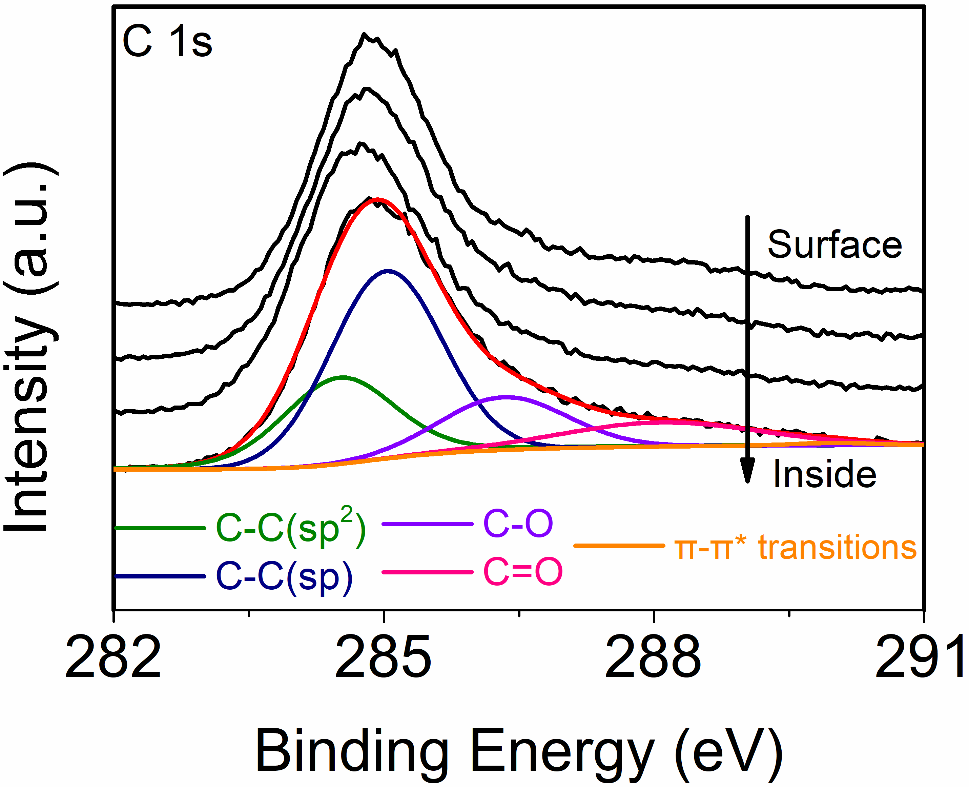


**Supplementary Figure S9.** High-resolution C 1s XPS spectra of Pd NPs/GDY recorded during the depth profiling experiments.


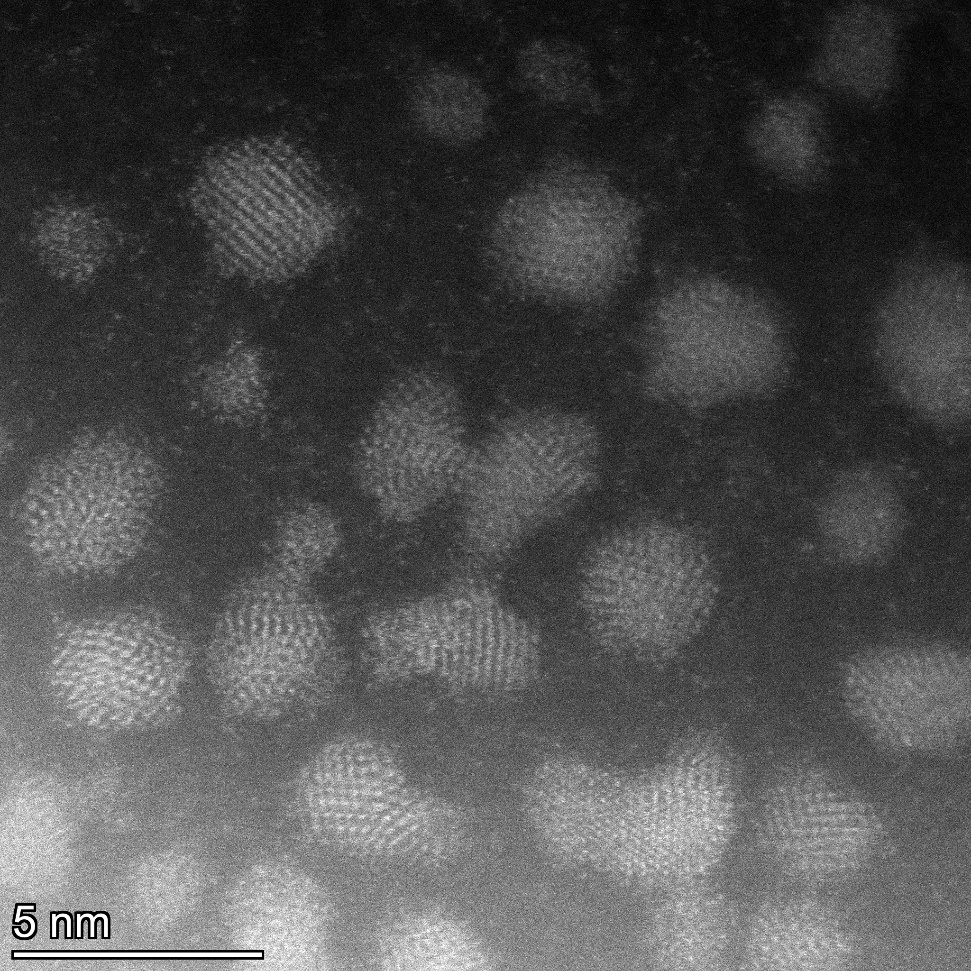


**Supplementary Figure S10.** HAADF-STEM image of Pd QDs/GDY.


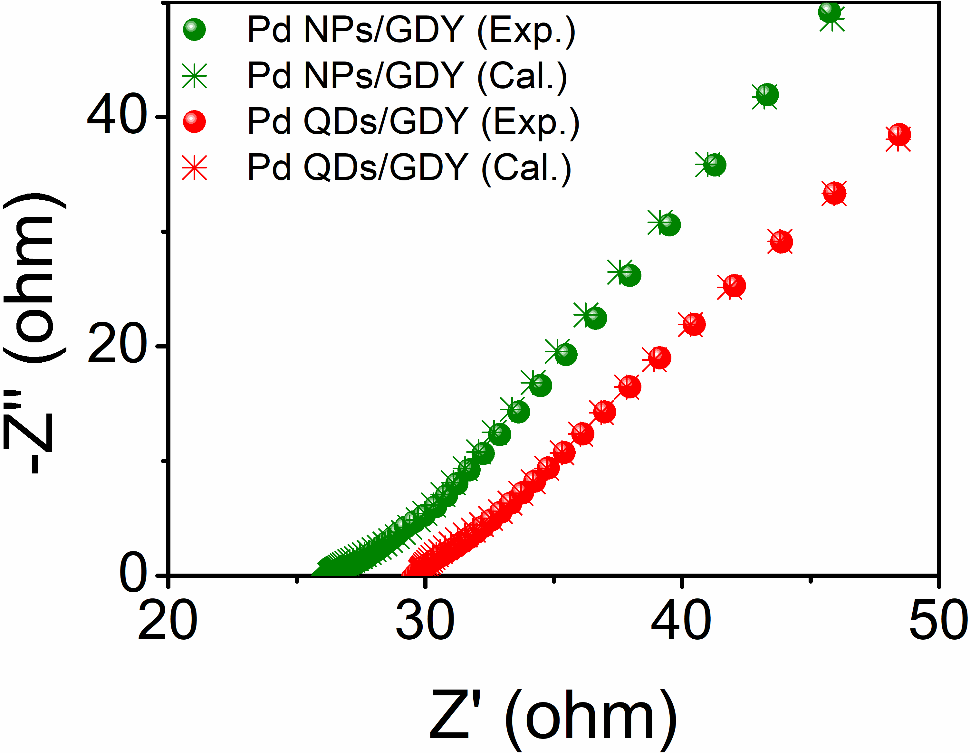


**Supplementary Figure S11.** Nyquist plots and corresponding fitting curves of Pd QDs/GDY and Pd NPs/GDY.


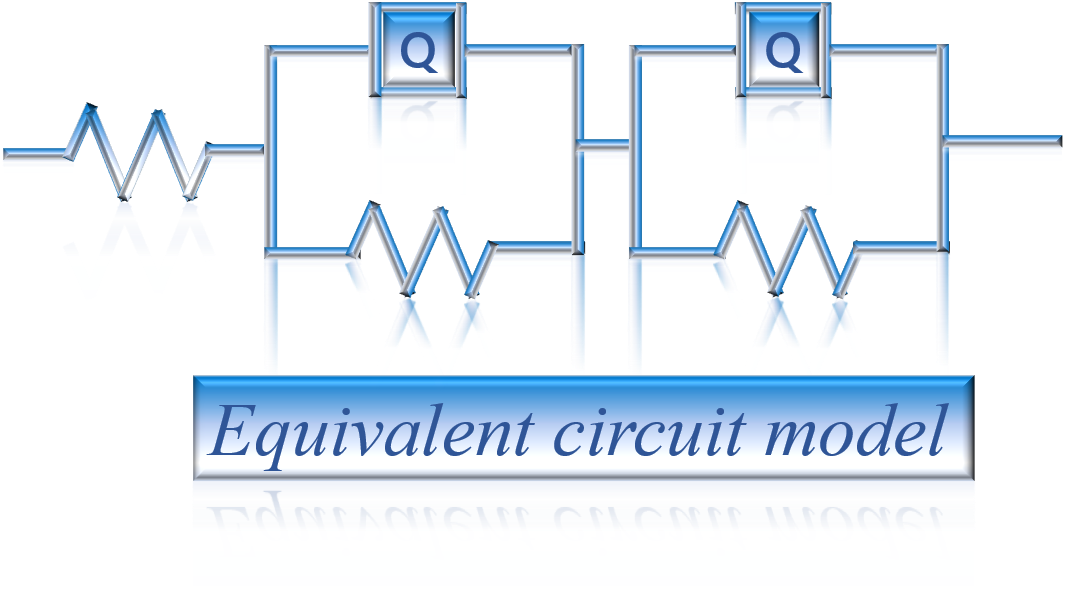


**Supplementary Figure S12.** Equivalent circuit model of Pd QDs/GDY and Pd NPs/GDY.


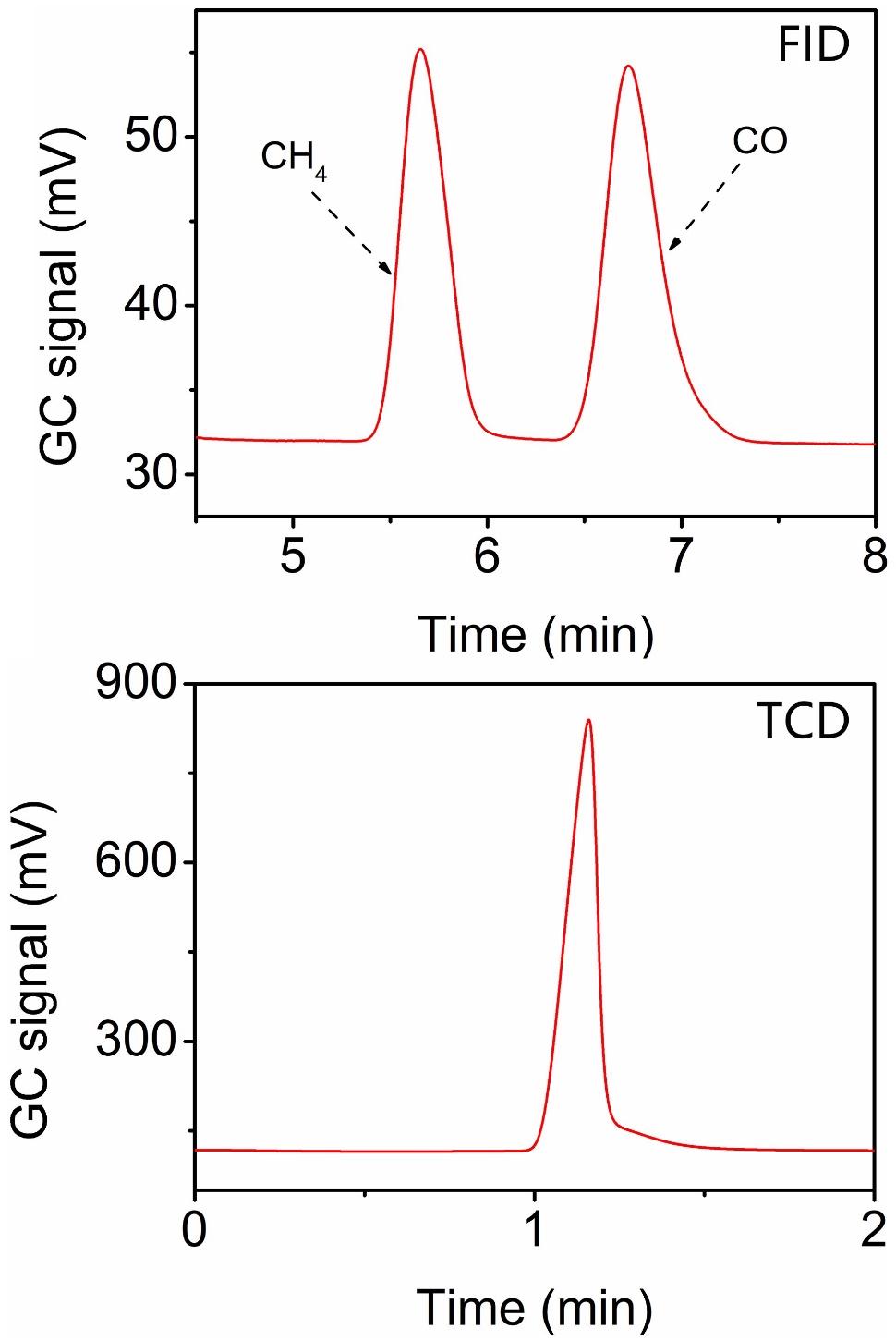


**Supplementary Figure S13.** Gas chromatogram data of external standard gas (CH_4_: 101 ppm, CO: 101 ppm and H_2_: 4.99%).


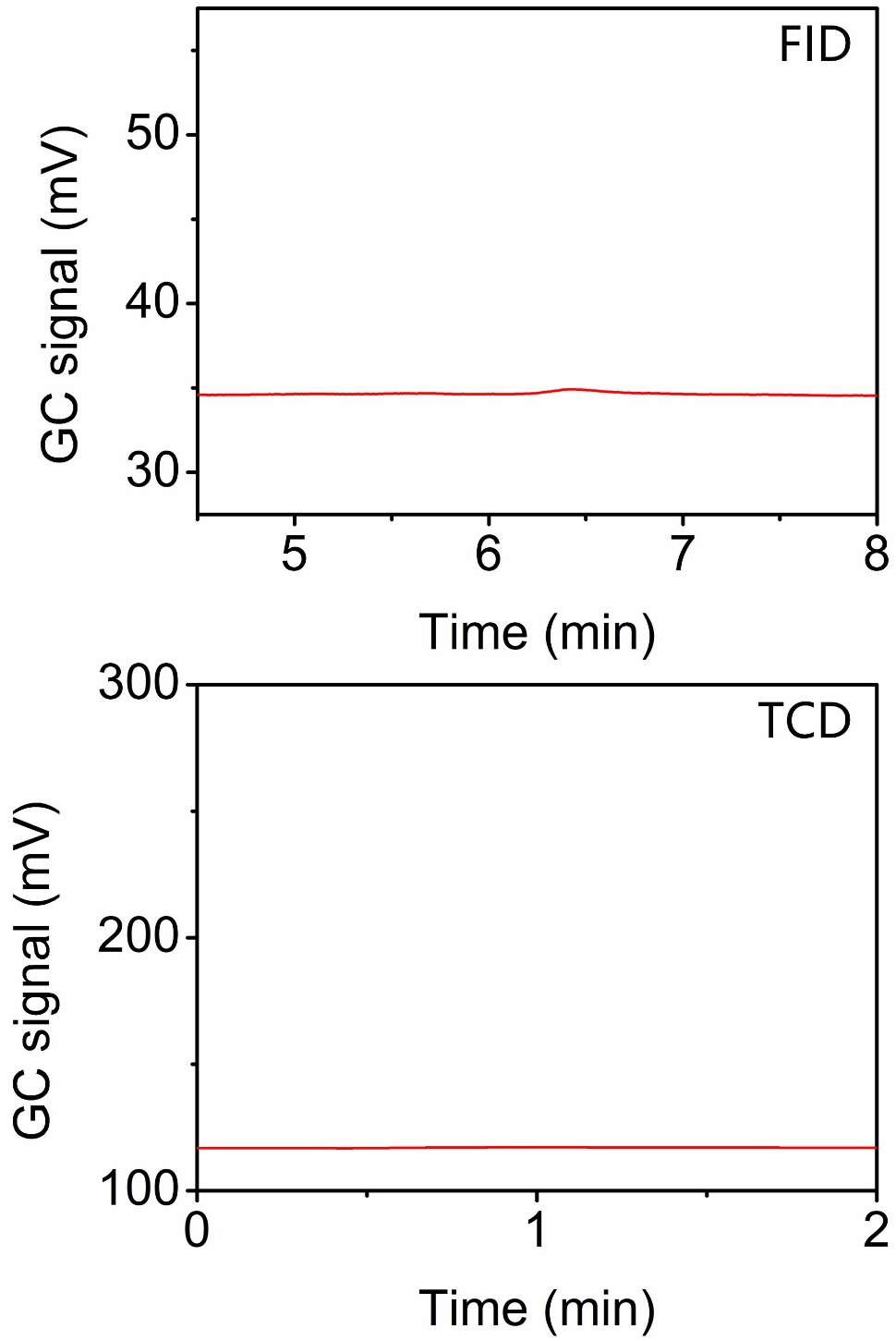


**Supplementary Figure S14.** Gas chromatogram data of high-purity carbon dioxide.


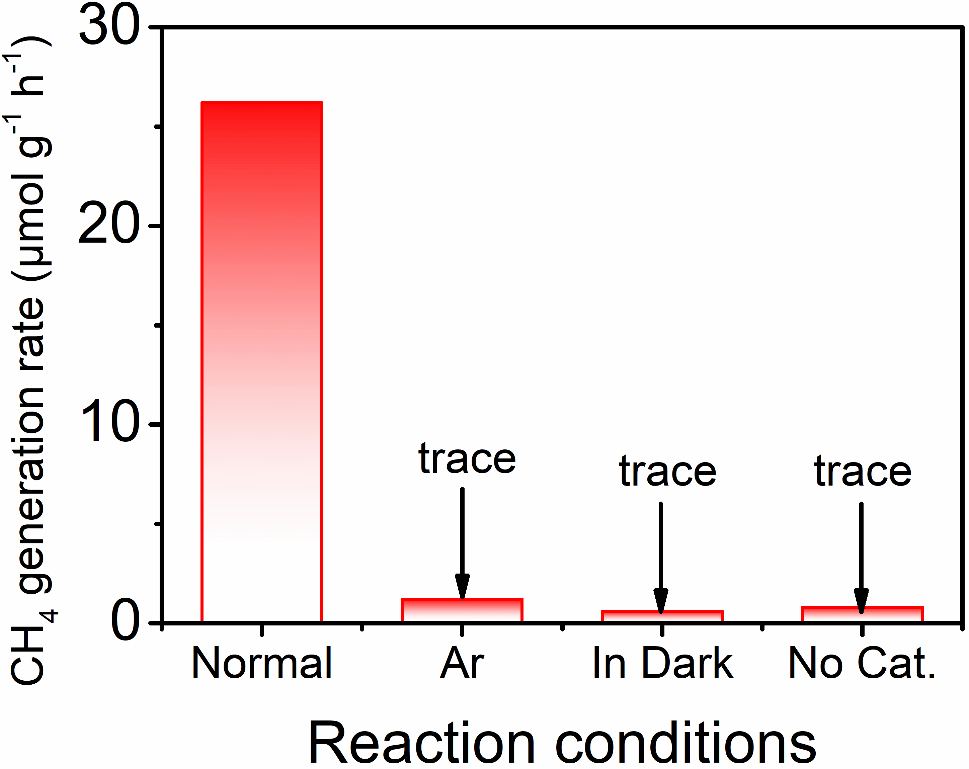


**Supplementary Figure S15.** Photocatalytic CH_4_ production of Pd QDs/GDY under different conditions.


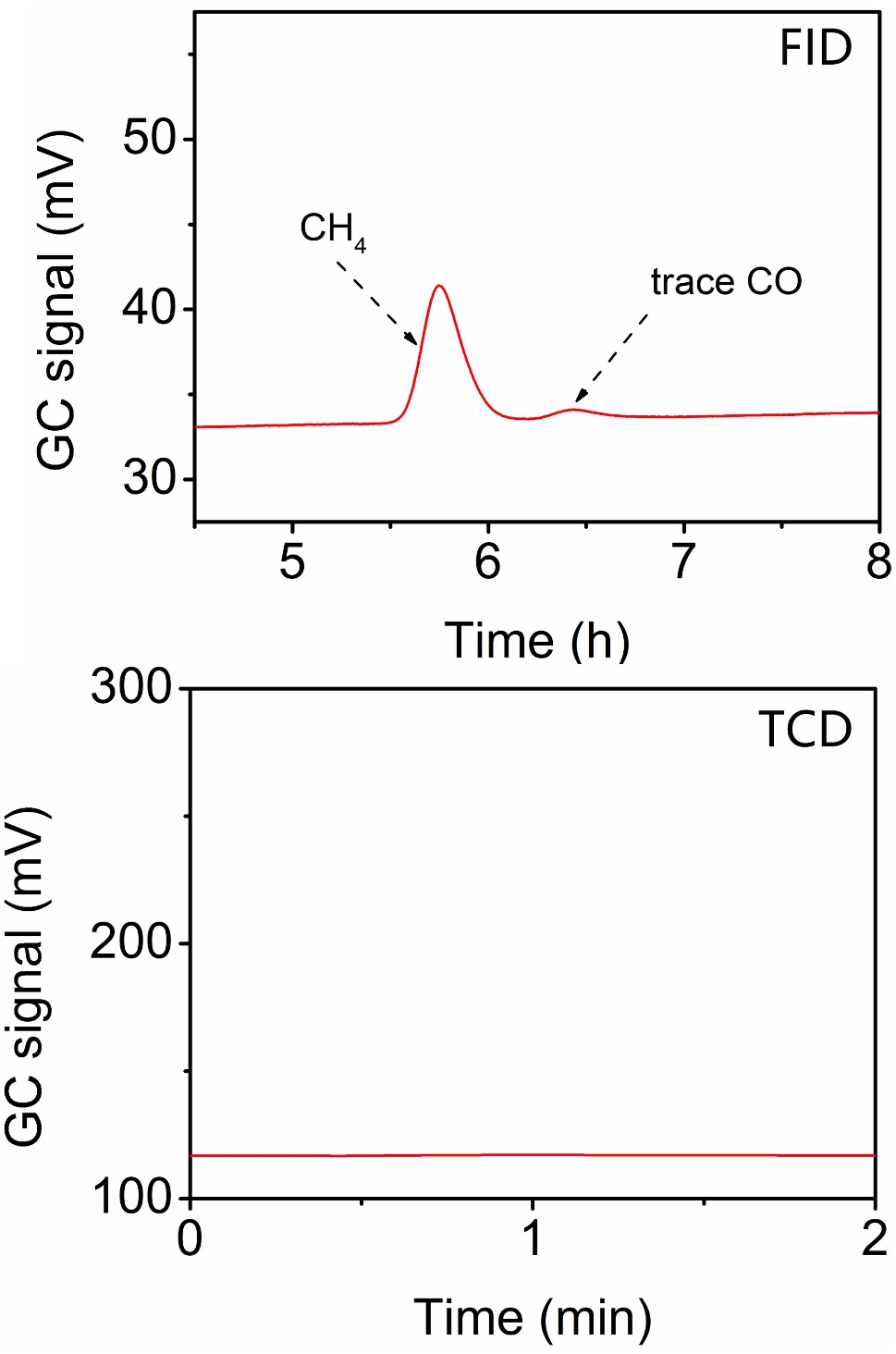


**Supplementary Figure S16.** Gas chromatogram data of gas-phase in photocatalytic reactor after 4h illumination.


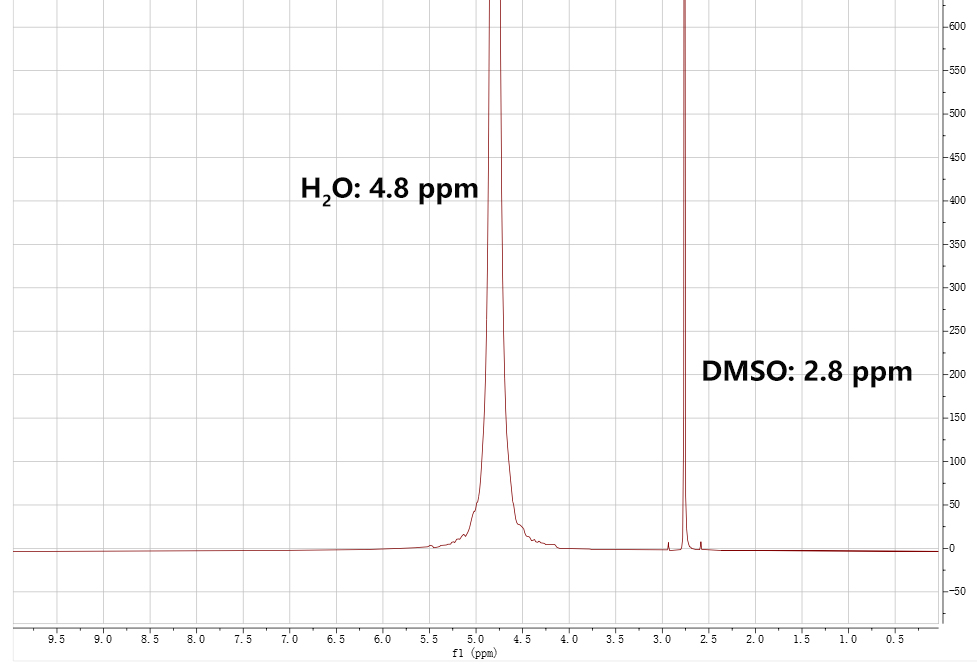


**Supplementary Figure S17.** NMR data of liquid-phase in photocatalytic reactor after 4h illumination.


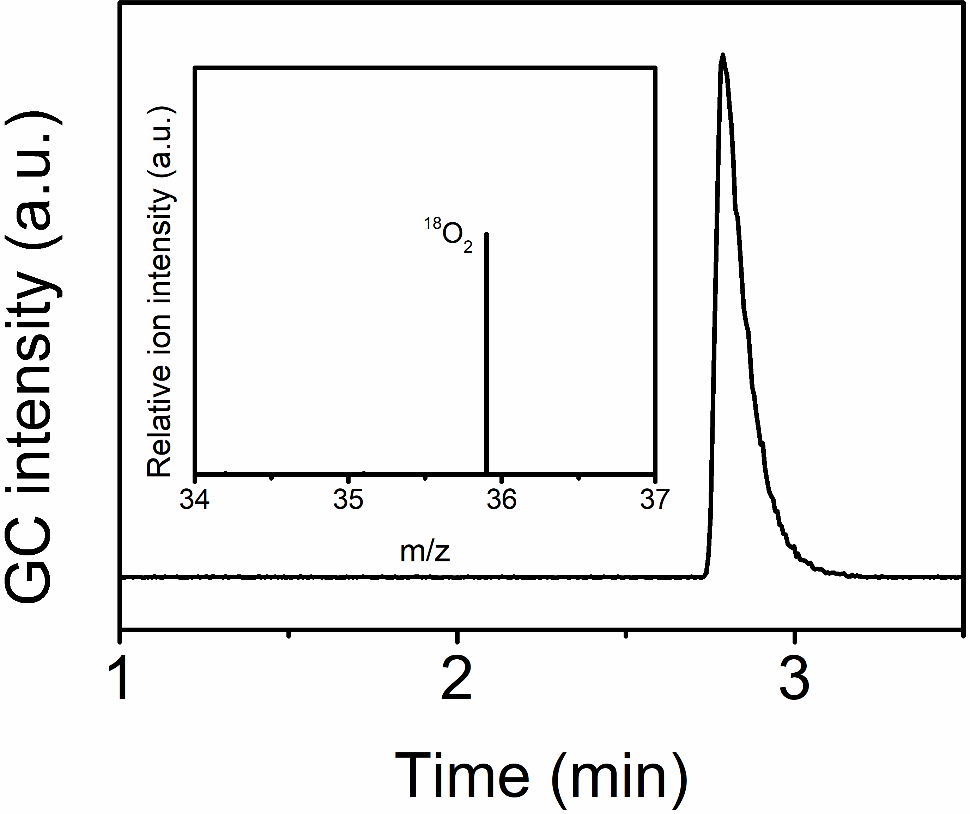


**Supplementary Figure S18.** MS results of ^18^O_2_ (m/z=35.9) converted from H_2_^18^O.


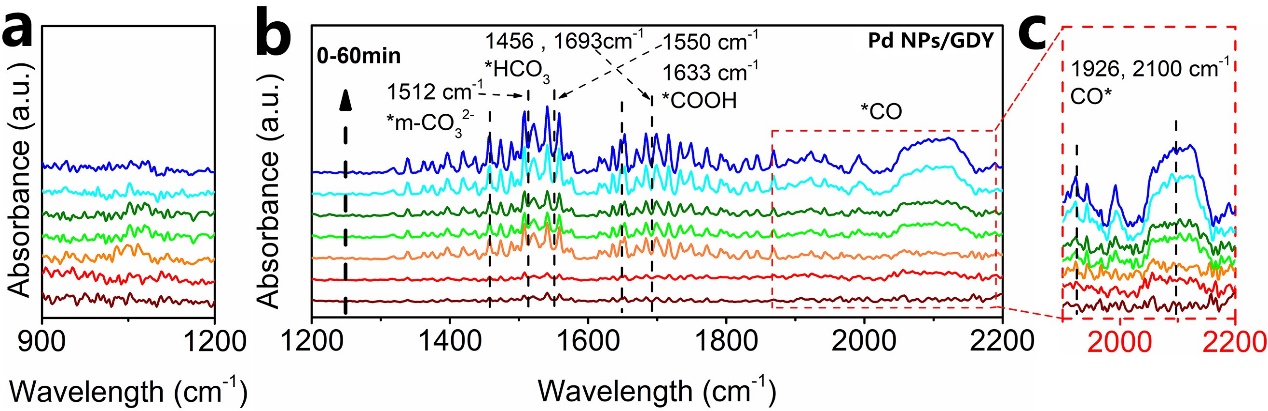


**Supplementary Figure S19.** In-situ DRIFTS spectroscopy of Pd NPs/GDY (a-c) during artificial photosynthesis.


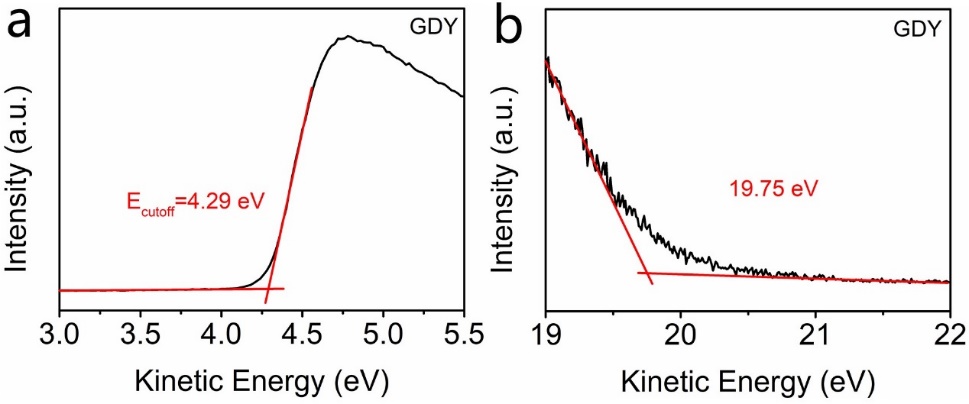


**Supplementary Figure S20.** UPS results of GDY.


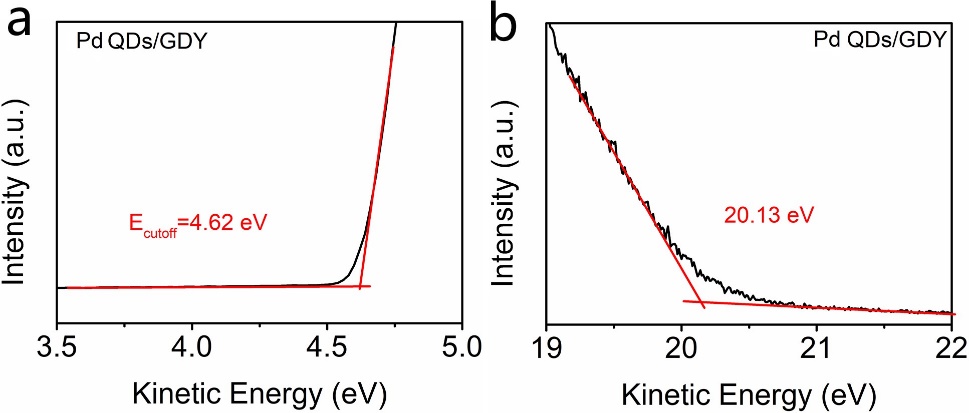


**Supplementary Figure S21.** UPS results of Pd QDs/GDY.


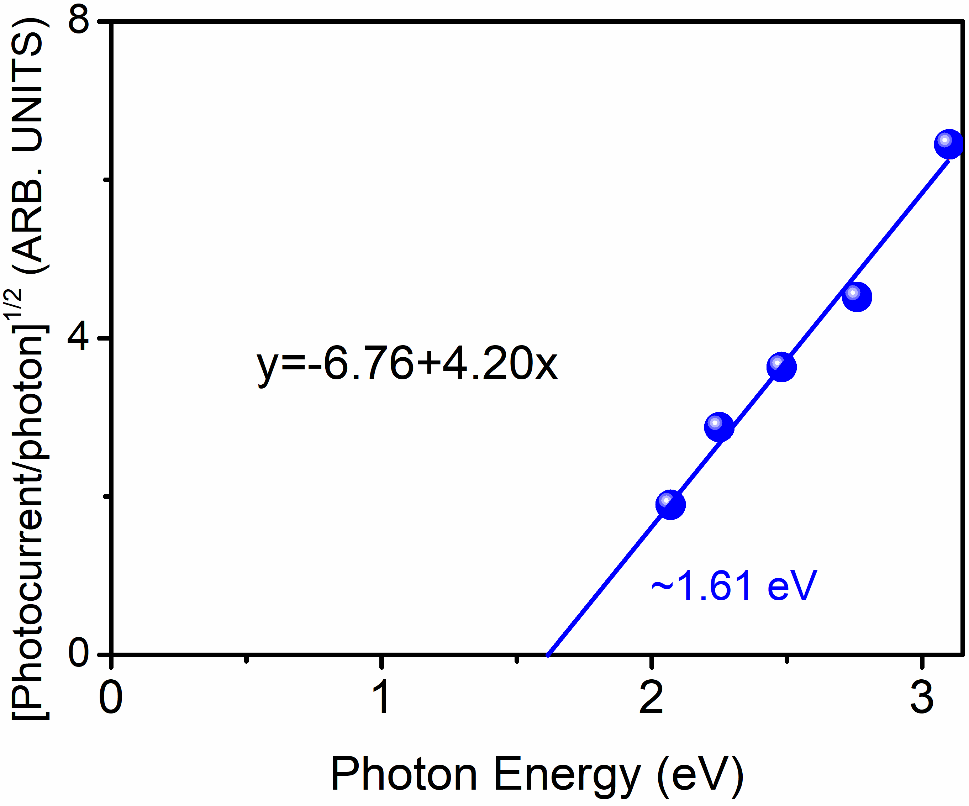


**Supplementary Figure S22.** Linear fitting of the normalized photocurrent to the photon energy of Pd NPs/GDY.

**3. Supplementary Tables**

**Supplementary Table S1.** XRD peaks and corresponding planes of Pd (PDF#46-1043).

| **Number** | **2 Theta (**°**)** | **Planes (**Å**)** | **(h k l)** |
| --- | --- | --- | --- |
| 1 | 40.12 | 2.22 | (1 1 1) |
| 2 | 46.66 | 1.95 | (2 0 0) |
| 3 | 68.12 | 1.38 | (2 2 0) |
| 4 | 82.10 | 1.17 | (3 1 1) |
| 5 | 86.62 | 1.12 | (2 2 2) |
| 6 | 104.75 | 0.97 | (4 0 0) |
| 7 | 119.33 | 0.89 | (3 3 1) |
| 8 | 124.63 | 0.87 | (4 2 0) |

**Supplementary Table S2.** Solid fluorescence lifetime fitting data.

|  | τ_1_ (ns) | τ_2_ (ns) | χ^2^ |
| --- | --- | --- | --- |
| GDY | 0.19 | 4.32 | 1.135 |
| Pd NPs/GDY | 0.17 | 4.93 | 1.080 |
| Pd QDs/GDY | 0.94 | 5.00 | 1.228 |

Supplementary Table S3. EIS parameters of the catalysts analyzed using Q(QR)(QR) equivalent model.

| Catalysts | Pd QDs/GDY | Pd NPs/GDY |
| --- | --- | --- |
| R_s_ (Ω) | 29.73 | 26.25 |
| CPE_1_ (S sec^n^) | 1.36×10^-4^ | 1.24×10^-3^ |
| Freq Power n_1_ | 0.774 | 0.813 |
| R_ct_ (Ω) | 573.4 | 1255 |
| CPE_2_ (S sec^n^) | 2.73×10^-5^ | 1.59×10^-3^ |
| Freq Power n_2_ | 1 | 0.706 |
| R’ (Ω) | 1.534 | 2.857 |

**Supplementary Table S4.** Comparison with other catalysts for photo-driving CO_2_ hydrogenation to CH_4_.

| Catalyst | Light source | Reaction medium | Products | Selectivity | Activity (μmol g^-1^ h^-1^) | Ref. |
| --- | --- | --- | --- | --- | --- | --- |
| Pd QDs/GDY | 300 W  Xe lamp | Gas-solid,  Water | CH_4_ | 98.5% | 26.2 | This work |
| BV/CoDAC | 150 W  Xe lamp | Gas-solid,  Water | CH_4_ | 61% | 19.7 | Angew. Chem. Int. Ed. 2022, 61, e202113044 |
| Pd_1+NPs_/C_3_N_4_ | 300 W  Xe lamp | Gas-solid,  Water | CH_4_ | 97.8% | 20.3 | Adv. Mater. 2022, 2200057. |
| Nb_2_O_5−x_ NFs | 300 W  Xe lamp | Gas-solid,  Water | CH_4_ | 64.8% | 19.5 | Adv. Mater. 2022, 2200756. |
| Au/TiO_2_/W_18_O_49_ | 300 W  Xe lamp | Gas-solid,  Water | CH_4_ | 93.3% | 35.55 | Adv. Mater. 2022, 34, 2109330. |
| STAO/TiO_2_ | 300 W  Xe lamp | Gas-solid,  Water | CH_4_ | 86.5% | 60.6 | Adv. Mater. 2022, 2109074. |
| CdG2 | 350 W  Xe lamp | Gas-solid,  Water | CH_4_ | 96.9% | 2.59 | Adv. Mater. 2019, 31, 1902868 |
| V_S_-CuIn_5_S_8_ | 300 W  Xe lamp filter | Gas-solid,  Water | CH_4_ | ~100% | 8.7 | Nat. Energy 2019, 4, 690-699. |
| V_o_-Zn-CoO | 300 W  Xe lamp | Gas-solid,  Water | CH_4_ | 63.8% | 26.8 | Adv. Funct. Mater. 2021, 2109336. |
| Cl-doped Cu_2_O | 350 W  Xe lamp | Gas-solid,  Water | CH_4_ | 47.3% | 0.39 | Nano Energy 2019, 60, 576-582. |
| Cu^2+^/GDYO | 300 W  Xe lamp | Gas-solid,  Water | CH_4_ | 87% | 11.86 | Sol. RRL 2021, 5, 2100200 |
| NH2-MIL-125(Ti) | 300 W  Xe | Gas-solid,  TEOA | CH4 | 32.9% | 1.01 | ACS Catal. 2021, 11, 650−658 |
| C-ZNNS60 | 350 W  Xe lamp | Gas-solid,  Water | CH_4_ | 43.8% | 1.14 | Nanoscale, 2020, 12, 7206–7213 |

**Supplementary Table S5.** Ultraviolet Photoelectron Spectrometer (UPS) results

|  | Au_Fermi_ (eV) | E_cutoff_ (eV) | W_F_ (eV) | HOMO (eV |
| --- | --- | --- | --- | --- |
| GDY | 21.22 | 4.29 | -4.29 | -5.76 |
| Pd QDs/GDY | 21.22 | 4.62 | -4.62 | -5.71 |
